# Case Report: an extremely rare case of giant dedifferentiated retroperitoneal liposarcoma

**DOI:** 10.3389/fonc.2025.1489833

**Published:** 2025-03-17

**Authors:** Huey Bing Chua, Rizuana Iqbal Hussain, Nordashima Abd Shukor, Xeng Inn Fam

**Affiliations:** ^1^ Urology Unit, Department of Surgery, Hospital Canselor Tuanku Muhriz, Universiti Kebangsaan Malaysia, Kuala Lumpur, Malaysia; ^2^ Department of Radiology, Faculty of Medicine, Universiti Kebangsaan Malaysia Medical Centre, Kuala Lumpur, Malaysia; ^3^ Department of Pathology, Faculty of Medicine, Universiti Kebangsaan Malaysia, Kuala Lumpur, Malaysia; ^4^ Urology Unit, Department of Surgery, Faculty of Medicine, Universiti Kebangsaan Malaysia, Kuala Lumpur, Malaysia

**Keywords:** liposarcoma, renal, retroperitoneum, surgery, case reports

## Abstract

Retroperitoneal liposarcoma, especially dedifferentiated liposarcoma (DDL), is a rare tumor type primarily affecting middle-aged and older adults in the retroperitoneum or proximal extremities. This case report highlights an exceptionally large retroperitoneal DDL that had enveloped the entire right kidney and had adhered to nearby tissues. Diagnosing retroperitoneal liposarcoma is challenging due to its asymptomatic nature until it reaches a substantial size. Imaging, particularly contrast-enhanced computed tomography (CECT), play a vital role in diagnosis, staging, and preoperative planning. Surgical resection, with the goal of R0 resection, remains the cornerstone of treatment, albeit this can be challenging due to tumor location. First-line treatment for advanced DDL involves anthracycline-based therapy. Eribulin and pazopanib show promise in second-line treatment. Ongoing clinical trials suggest a shift towards multimodal therapy. This case report reports the largest retroperitoneal liposarcoma and underscores the complexity of managing retroperitoneal DDL.

## Introduction

Liposarcoma is also known as soft tissue sarcoma (STS) (1). Liposarcomas represent the predominant subtype of sarcomas that emerge within the retroperitoneum, constituting approximately 41% of cases (2). Liposarcoma appears to arise from adipocytes (fat cells) and is most common in the extremities (52%), followed by the retroperitoneum (12-40%) and perirenal fat (35%) (2). Retroperitoneal liposarcoma is very rare, with an incidence of 2.5 per million population, and is usually asymptomatic until they are large enough to compress the adjacent structures (3). Although biopsy is the gold standard for diagnosis, imaging is accepted as a modality for diagnosis and staging; biopsy is not usually required (4). Surgery is the treatment of choice for retroperitoneal liposarcoma with or without resecting the surrounding structures. The 5-year survival rate for a histologically well-differentiated liposarcoma is 83%, while for a dedifferentiated tumor, it is approximately 20% (5). Herein, we present a case of an extremely large retroperitoneal dedifferentiated liposarcoma (DDL), the largest retroperitoneal liposarcoma reported in the literature to date.

## Case report

**Figure 1:**
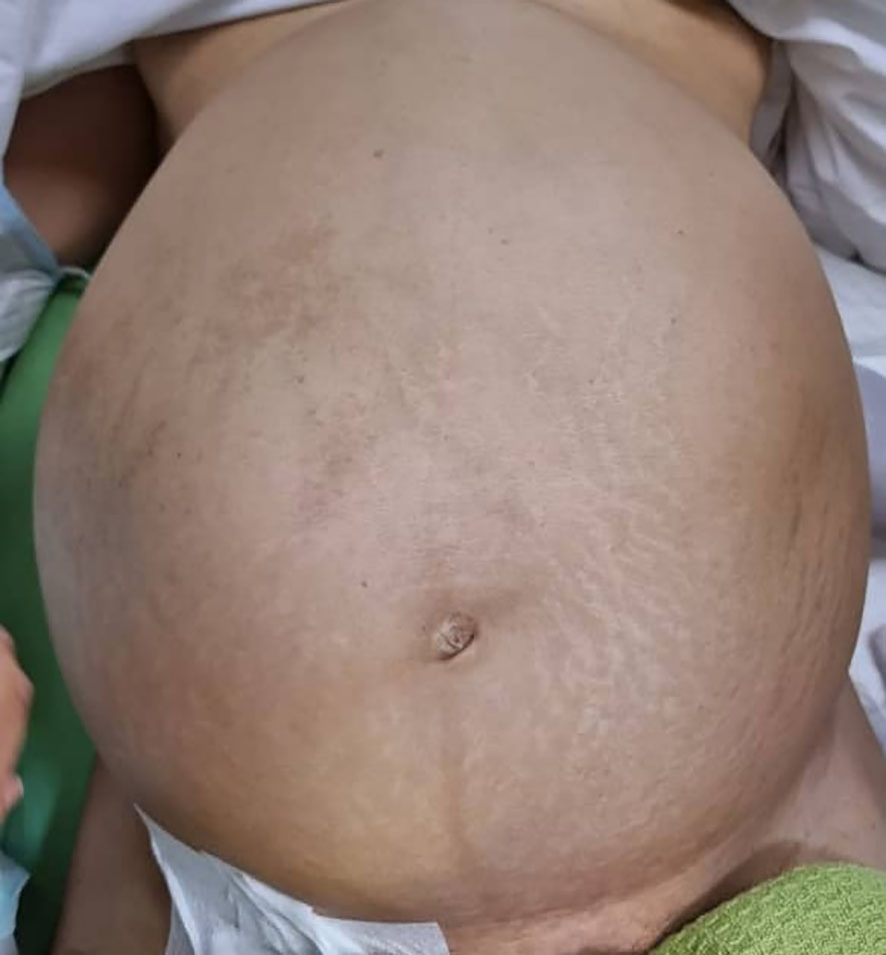
Clinical examination showed a huge abdominal mass with a midline shift.

**Figure 2:**
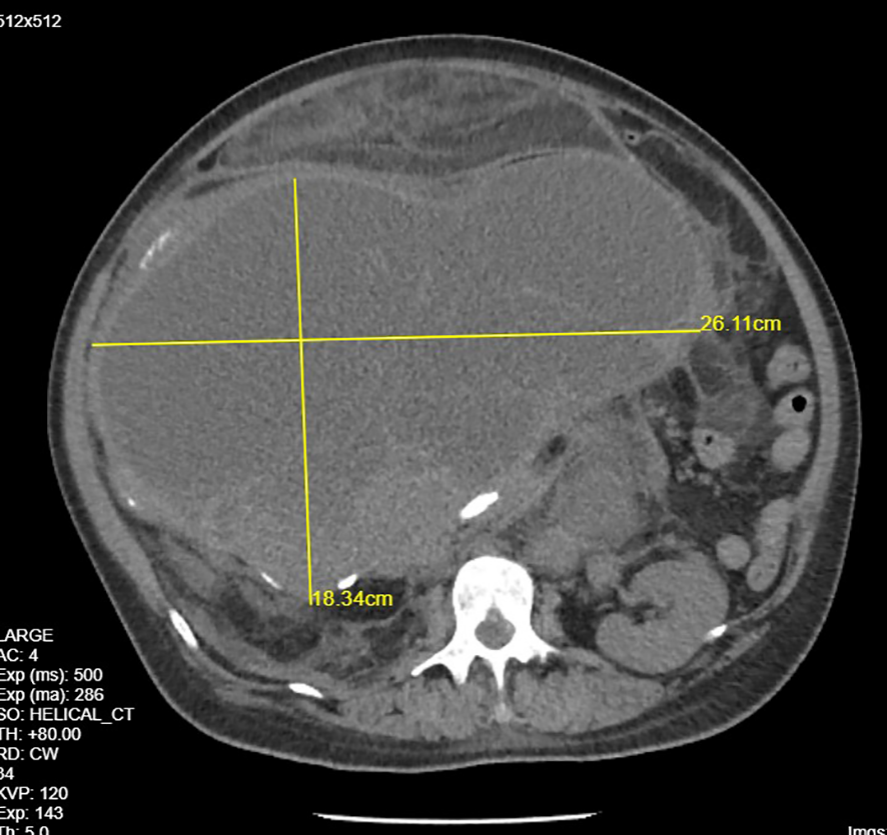
CECT renal scan of the right kidney showing a huge renal mass.

**Figure 3:**
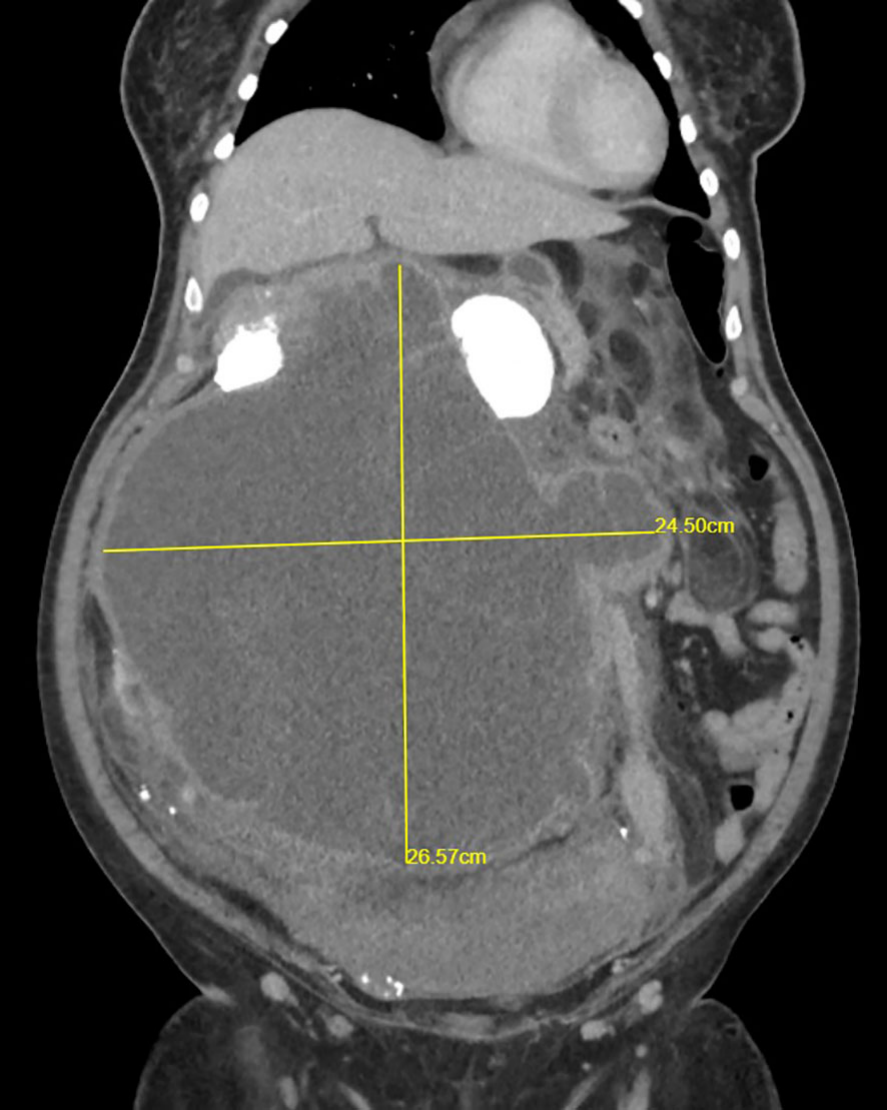
CECT renal scan of the right kidney showing a huge renal mass with no clear plane with the right side of the colon.

A 44-year-old woman with no comorbidity was referred to a urology clinic with progressive abdominal distension over the last 2 years. Her vital signs were stable and her systemic reviews were normal. The abdominal examination revealed a distended abdomen with a firm mass that was irregular, non-tender, and did not move with respiration. It was extended from the right subcostal margin to the right iliac fossa, measuring 20 x 25 x 30 cm (AP x W x CC) (**Figure 1**), and crossed the midline. Baseline laboratory investigations including renal function tests were normal. The contrast-enhanced computed tomography (CECT) renal scan showed a massive solid cystic right kidney mass measuring 16.9 x 25.6 x 26.5 cm (AP x W x CC) with a fat component and multiple enlarged intraabdominal lymph nodes with coarse calcification (**Figure 2**). The mass did not have any vascular invasion. There was no clear plane between the mass and the right side of the colon as shown in **Figure 3**. The renal veins were patent without any thrombosis. The diagnosis was confirmed, and an open approach was used to perform an *en-bloc* surgical resection of the giant mass.

**Figure 4:**
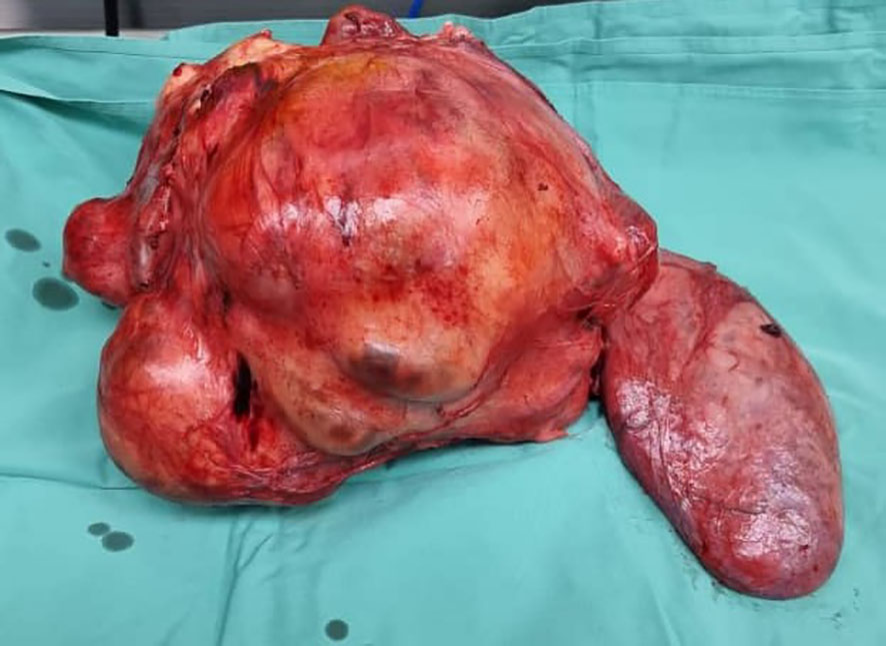
Resected retroperitoneal liposarcoma.

Intraoperatively, a giant right kidney tumor infiltrating into the right adrenal gland with no clear demarcation from both the right common iliac vein and the inferior vena cava (IVC) was revealed. The tumor was shaved off from the IVC and a right common iliac vein defect was repaired (**Figure 4**). The tumor was also densely adherent to the small bowel and the ascending colon. Careful adhesiolysis was performed, successfully freeing the small bowel from the tumor. However, after mobilizing the ascending colon, it appeared thin and discolored, requiring a right hemicolectomy and the creation of a double barrel stoma with the assistance of the colorectal team. The giant mass was successfully resected *en bloc*.

**Figure 5:**
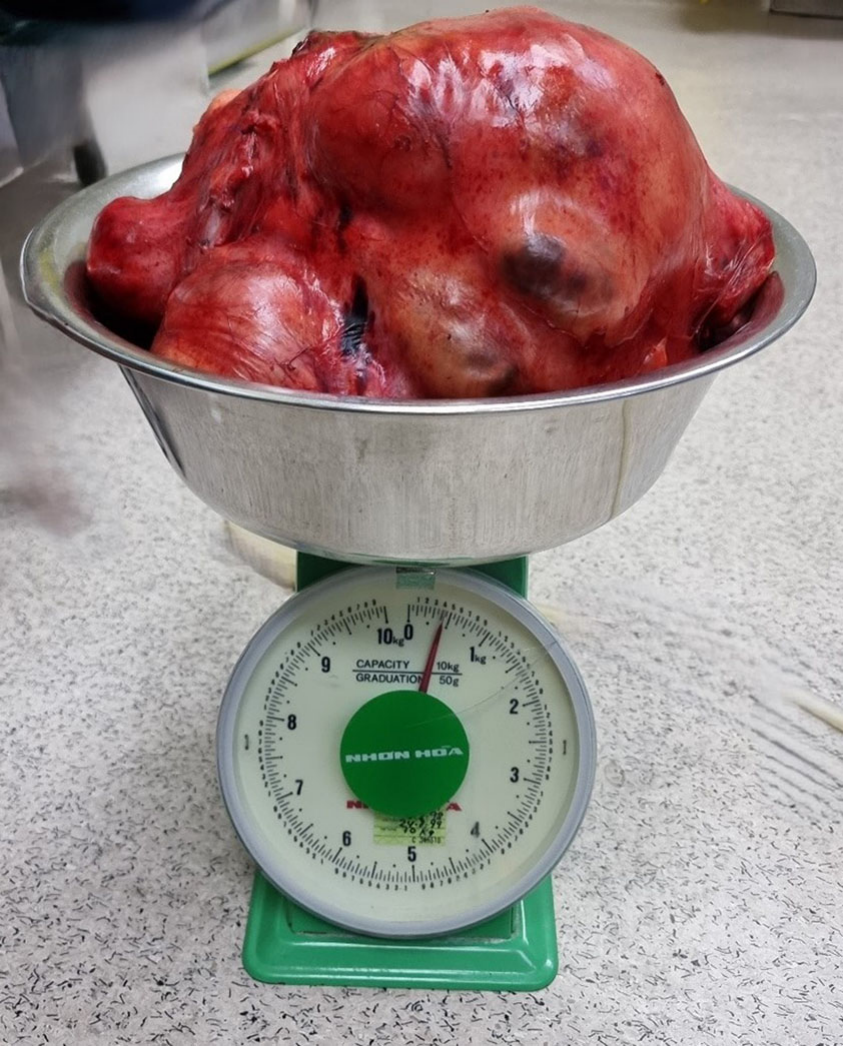
Retroperitoneal liposarcoma weighing 10.4kg.

**Figure 6:**
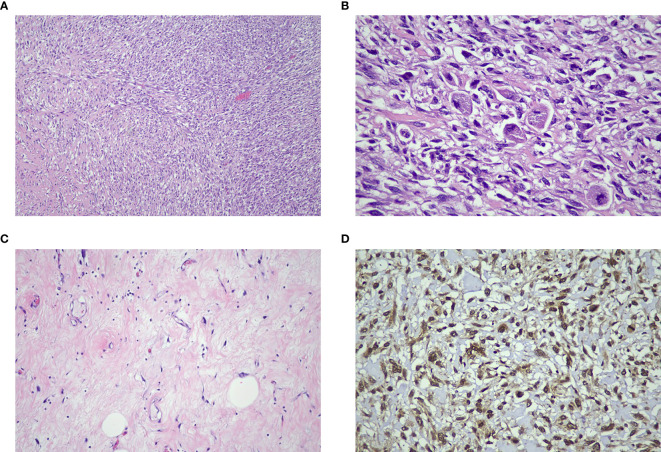
**(A)** The tumor consists of high-grade malignant cells arranged in sheets and interlacing fascicles with a herring-bone pattern (H&E x 100 mag.). **(B)** Some cells display enlarged spindle-shaped pleomorphic hyperchromatic nuclei and scanty cytoplasm (H&E x 400 mag.). **(C)** Focal areas of well-differentiated adipocytes with atypical stroma cells were identified (H&E x 200 mag.). **(D)** The diffuse immune expression positivity towards MDM2 confirmed the diagnosis of dedifferentiated liposarcoma (IHC x400 mag.).

On gross examination, the retroperitoneal tumor measured 30x50x25cm and weighed 10.4kg (**Figure 5**). The macroscopic examination showed a large multilobulated solid cystic tumor involving almost the entire kidney and renal sinus fat. Furthermore, the microscopic examination of the resected specimen showed the kidney tumor was composed of low- and high-grade malignant cells with a well-differentiated liposarcoma focal area and no lymphovascular invasion was found. Immunohistochemistry studies showed the malignant cells were diffusely positive for murine double minute 2 (MDM2) with weak positivity for smooth muscle antibody (SMA). They are negative for cytokeratin AE1/AE2 (CKAE1/AE2), S100 protein, desmin, and CD34 suggestive of dedifferentiated liposarcoma with clear margin (**Figure 6**).

Her post-operative recovery was complicated by surgical site infection and retroperitoneal collection. We proceeded with a pigtail drainage and she was subsequently discharged. A follow-up CECT thorax and abdomen scan conducted 2 months later revealed a recurrence of liposarcoma in the right abdominal region, prompting the initiation of intravenous doxorubicin at a dose of 60mg. A follow-up CECT thorax and abdomen scan performed 9 months later showed a smaller retroperitoneal liposarcoma mass.

## Discussion

Overall, retroperitoneal tumors represent 1% of all malignancies and liposarcoma is the most common to occur in the retroperitoneum (41%) (2). Retroperitoneal liposarcoma has an incidence of 2.5 per million populations (6). Patients who present with retroperitoneal liposarcoma are typically in their mid-fifties. However, it is worth noting that Retroperitoneal Sarcoma (RPS) cases have been reported across all age groups, spanning from 2 to 98 years old (7). The large area within the retroperitoneal space allows for the expansion and growth of a tumor. Therefore, tumors in this location tend to be asymptomatic in 80% of the population or present with non-specific symptoms. The retroperitoneal liposarcoma in our case weighed 10.4kg with dimensions of 30x50x25cm and is the largest retroperitoneal liposarcoma reported in the literature to date.

According to the WHO, there are four histological subtypes for liposarcoma: 1) well-differentiated 2) dedifferentiated, 3) myxoid/round cell, and 4) pleomorphic. The well-differentiated subtype is the most common sarcoma arising in the retroperitoneum (8). DDL is defined as the transition from well-differentiated liposarcoma (WDL) or atypical lipomatous tumor (ALT) to non-lipogenic sarcoma, which arises mostly in the retroperitoneum and deep soft tissue of proximal extremities (9). The incidence of DDL is less than 0.1 per 1,000,000 each year (10), making our case extremely rare. DDL is characterized by a supernumerary ring and giant marker chromosomes, both of which contain amplified sequences of 12q13-15 including MDM2 and cyclin-dependent kinase 4 (CDK4) cell cycle oncogenes. The overall 5- and 10-year overall survival (OS) probabilities for DDL in were 51.5% and 34.8%, respectively, with a median OS of 63.6 months (11).

DDL are aggressive mesenchymal tumors known for their genomic amplification of the MDM2 oncogene (9). While the MDM2 amplification is well established as a diagnostic criterion for DDL, the variability and clinical implications of the extent of MDM2 amplification have not yet been fully elucidated. There are multiple therapeutic approaches that aim to target MDM2 and CDK4 activity with the aim to restore the intrinsic tumor suppressor cellular response and to terminate oncogenesis. However, current understanding of the molecular mechanisms involved in well differentiated liposarcoma (WDLPS) and dedifferentiated liposarcoma (DDLPS) pathology is limited (12). Patients with DDLPS in advanced stages typically experience poor outcomes, with limited therapeutic options and a lack of validated biomarkers for prognosis or chemotherapy selection.

In the case of liposarcoma, imaging remains the primary diagnostic tool. CECT scans are the most used modality whereas MRI is debated as an equally efficacious technique but lacks large-scale comparisons (2). On a CT scan, retroperitoneal liposarcoma usually appears as a large, encapsulated mass containing variable amounts of fatty and soft tissue components (1). MRI is important for the diagnosis of liposarcoma invasion of the abdominal aorta or inferior vena cava. Although biopsy is the gold standard in diagnosing liposarcoma, it is not strongly recommended unless the patient is physically unfit for an operation or needs preoperative chemo/radiotherapy (7).

Surgical resection remains the only potentially curative therapy for retroperitoneal liposarcoma with a resectability rate of 100% and 87% for local recurrence. Successful complete resection of retroperitoneal liposarcoma may increase the 5-year survival rate from 16.7% to 58% (2). Most clinicians suggest R0 resection (complete resection with a microscopically negative margin), although R1 resection (microscopically positive margin) is also accepted at the cost of a high recurrence rate. Wide resection is the standard treatment for local disease. R0 resection is achievable for DDL located in the extremities but is more challenging for retroperitoneal tumors (13). In surgical practice, the selection of which procedure is suitable for an individual patient must be based on tumor location, size, stage, relationship with surrounding neurovascular and bone elements, and functional and cosmetic requirements. Nevertheless, a complete R0 resection should be the goal during surgery and, to achieve this, an *en-bloc* resection along with adjacent structures is sometimes needed (13).

A multidisciplinary approach is required in treating retroperitoneal liposarcoma. Preoperative radiotherapy and chemotherapy may be considered in cases when total resection is not possible or when dealing with high-grade sarcoma. However, it is important to note that preoperative radiation is not currently recommended for resectable retroperitoneal liposarcoma (14). In a recent randomized trial (EORTC-62092, STRASS), the combination of radiotherapy and surgery did not show a clear benefit in terms of recurrence-free survival when compared to surgery alone (15).

The development of unresectable local and/or metastatic DDL has a poor prognosis. Locoregional recurrence of WDL/DDL represents the main contributing factor of mortality and disease burden, with 40% to 80% of patients experiencing recurrence after surgery (16). In this section, we provide an overview of the latest advancements in the treatment of advanced DDL and shed light on ongoing and prospective research in this field.

Like various other subtypes of STSs, the use of anthracycline-based therapy is considered a standard initial treatment approach for advanced DDL (17). In the EORTC-62012 phase 3 trial, a *post hoc* subgroup analysis revealed that there was no enhancement in the objective response rate (ORR) or OS among patients with liposarcoma who received the combination therapy of doxorubicin and ifosfamide when compared to those who received doxorubicin alone (18).

Several retrospective studies have been conducted regarding the role of anthracycline-based treatment in patients with advanced DDL (19). In the largest multicenter study, which consisted of 208 patients, 171 (82%) had DDL. Approximately 82% of patients received anthracycline-based therapy. Among 167 patients, OR was observed in 21 patients (12%) and the median OS was 15.2 months. Another extensive single-center study also showed an ORR of 20%. Considering the collective evidence, it is reasonable to suggest that anthracycline-based regimens can be regarded as an appropriate first-line treatment for advanced DDL (20).

Eribulin is presently authorized for the treatment of individuals with unresectable or metastatic liposarcoma who have previously undergone anthracycline-based therapy. This approval was granted based on findings from a phase 3 trial involving 452 patients with advanced liposarcoma or leiomyosarcoma, which was conducted at multiple centers and was open-label in nature (20). Eribulin is anticipated to lead to an enhancement in OS among patients with advanced DDL (21).

Pazopanib has received approval in several countries for the treatment of STSs in second or later-line settings among patients (22). Other multi-targeted tyrosine kinase inhibitors (TKIs) such as sunitinib, regorafenib, and anlotinib have also undergone investigation in phase 2 trials for the treatment of advanced STSs, which include liposarcoma. However, none of these TKIs are currently authorized for use specifically in liposarcoma (23–25).

Immunotherapy approaches have been tested in DDL, showing promising responses or durable disease control in a small population, though overall efficacy remains limited. For instance, the phase II SARC028 trial, which assessed pembrolizumab in soft tissue and bone sarcomas, included 10 patients with DDL. Of these, two patients (20%) achieved a partial response (PR), prompting the addition of a further 30 DDL patients. Across the 39 patients evaluated, the ORR was 10% and median progression-free survival (mPFS) was 2 months, indicating that pembrolizumab monotherapy has limited activity in DDL. Pembrolizumab or nivolumab (with or without ipilimumab) are listed in the National Comprehensive Cancer Network (NCCN) guidelines for subsequent lines of treatment for advanced or metastatic disease. Due to the limited options in systemic treatment, immune checkpoint inhibitor treatment is incorporated after approved chemotherapies (26).

Novel therapies targeting the unique molecular features of DDL are actively being explored, with promising results in some cases. DDL is characterized by 12q13-15 amplification, which leads to overexpression of CDK4 and MDM2.

CDK4/6 inhibitors, particularly palbociclib, have been tested in DDL. While two patients showed a partial response in phase II trials, the overall activity was limited, with a mPFS of only 18 weeks. More potent inhibitors, such as abemaciclib, showed slightly better results, with a 10% response rate and six patients maintaining disease control for over 2 years. Ongoing trials, such as SARC041 (NCT04967521), are further evaluating abemaciclib in DDL, and preclinical studies suggest CDK4/6 inhibition may alter the tumor immune microenvironment (27–29).

An MDM2 inhibitor, an E3 ligase that regulates p53, is another key target in DDL. Although the phase III MANTRA trial of milademetan, an MDM2 inhibitor, failed to meet its primary endpoint, preclinical findings suggest that MDM2 amplification and tumor heterogeneity may contribute to resistance. On a more promising note, the MDM2 inhibitor brigimadlin has shown encouraging results in a phase Ib trial, with a median PFS of 8.1 months and some durable responses. A phase II/III trial (Brightline-1) is now comparing brigimadlin to doxorubicin in advanced DDL (30, 31).

Given the relatively low response to PD-1 inhibitors alone in DDL, combining them with other therapies, such as CDK4/6 inhibitors, is being investigated. A phase II study combining the PD-1 inhibitor retifanlimab with palbociclib demonstrated an ORR of 14.3%. Another ongoing trial is exploring palbociclib in combination with the PD-1 inhibitor cemiplimab (32, 33).

In summary, DDL treatment, especially in advanced DDL, is evolving with the investigation of these novel agents, and further study is required on DDL biology to improve drug development for DDL.

When managing retroperitoneal liposarcoma in women of reproductive age, fertility-sparing treatment is crucial. This approach encompasses both surgical intervention and adjuvant therapies, highlighting the importance of fertility preservation in cancer care. Establishing a multidisciplinary team is essential to achieve both oncological control and the preservation of fertility. When considering treatment, it is vital to assess various prognostic factors, including tumor size, stage, grade, and histopathological type. During surgical procedures, meticulous care should be taken to preserve blood vessels, nerves, and healthy pelvic organs. Additionally, laparoscopic transposition prior to pelvic radiation therapy should be considered for women of childbearing age, as documented in the literature. Cryopreservation of ovarian tissue involves the removal and storage of ovarian fragments to protect against potential reproductive damage from cancer treatments. This technique serves a dual purpose: it can act as an oocyte reservoir for future orthotopic transplantation, helping to restore ovulatory function if compromised by gonadotoxic chemotherapy (34). This patient had completed her family, and fertility was not a concerning issue for her.

## Conclusion

Retroperitoneal liposarcoma is a rare tumor while DDL is the rarest subtype. Complete surgical resection remains the primary treatment choice providing the best prognosis. A multidisciplinary team should be formed to ensure optimal surgical planning and oncological treatment. Fertility preservation and oncofertility should be considered when managing giant non-gynecological malignancies in women of childbearing age. This case report highlights an exceptionally rare and large DDL, the largest of its kind reported to date. The case underscores the challenges of diagnosing and treating advanced DDL, emphasizing the importance of early detection and intervention. The patient’s management required a multidisciplinary approach, including complex surgical resection and careful postoperative care, demonstrating the need for a comprehensive strategy in such cases. Molecular insights into the tumor’s amplification of MDM2 and CDK4 suggest potential therapeutic targets for novel treatments such as CDK4/6 inhibitors and MDM2 inhibitors, which are currently under investigation. This case also highlights the role of early imaging in diagnosing large tumors before they become inoperable and the importance of personalized treatment plans based on tumor characteristics. Furthermore, fertility preservation and oncofertility should be considered when managing giant non-gynecological malignancies in women of childbearing age.

## Data Availability

The original contributions presented in the study are included in the article/supplementary material. Further inquiries can be directed to the corresponding author.
